# Effect of Sarcopenia on Functional Recovery in Acute Stroke Patients Admitted for Standard Rehabilitation Program

**DOI:** 10.3390/medicina60101716

**Published:** 2024-10-20

**Authors:** So-Yeong Kim, Woon-Su Cho, Chi-Bok Park, Byeong-Geun Kim

**Affiliations:** 1 Department of Physical Therapy, Graduate School, Nambu University, Gwangju 62271, Republic of Korea; 2 Rehabilitation Center, Gwangju 365 Rehabilitation Hospital, Gwangju 62232, Republic of Korea; 3 Department of Physical Therapy, Nambu University, Gwangju 62271, Republic of Korea

**Keywords:** acute, stroke, sarcopenia, convalescent rehabilitation

## Abstract

*Background and Objectives*: Sarcopenia is a significant concern in stroke rehabilitation, with a high prevalence reported in acute stroke patients. This study examines the effect of sarcopenia on rehabilitation outcomes in acute stroke patients. *Materials and Methods*: This study was conducted with acute stroke patients admitted within 90 days of onset to the rehabilitation hospital. Participants were divided into a stroke with sarcopenia group and a stroke without sarcopenia group. Evaluations were conducted at baseline, 4 weeks, and 8 weeks, including the following assessments: manual muscle testing (MMT), Berg Balance Scale (BBS), functional ambulation category (FAC), and Modified Barthel Index (MBI). Both groups received an identical rehabilitation program for 8 weeks. *Results*: Significant within-group improvements were observed in both groups across all measures (*p* < 0.05). However, the stroke with sarcopenia group showed significantly less improvement in MMT, BBS, FAC, and MBI compared to the stroke without sarcopenia group at both 4 and 8 weeks (*p* < 0.05). *Conclusions*: These results underscore the significant impact of sarcopenia on functional recovery in stroke patients, despite both groups receiving identical rehabilitation programs. The presence of sarcopenia was a critical predictor of poorer outcomes in muscle strength, balance, ambulation, and activities of daily living. Given these findings, specific rehabilitation strategies targeting sarcopenia are needed to improve recovery in stroke patients. Future research should include larger sample sizes, longer follow-ups, and sarcopenic patient-specific rehabilitation programs.

## 1. Introduction

Survivors of stroke, caused by either cerebral infarction or cerebral hemorrhage, experience various sequelae [1]. A majority of stroke survivors suffer from hemiplegia, resulting in paralysis of one side of the body [2]. Hemiplegia leads to an imbalance in bilateral muscle strength, negatively impacting functional activities such as gait and activities of daily living [3,4]. Several studies have reported the presence and impact of sarcopenia in stroke patients. Specifically, sarcopenia in stroke patients has been associated with older age, severe stroke, low body mass index, reduced calf circumference on the non-paretic side, impaired swallowing function, and poor functional outcomes [5,6,7]. Recently, the prevalence of sarcopenia in stroke patients admitted to rehabilitation hospitals during the recovery phase has been reported to be as high as 51% [8], highlighting the importance of preventing and managing sarcopenia in stroke rehabilitation.

Sarcopenia is particularly important in stroke patients due to its profound impact on the rehabilitation process. Studies have shown that sarcopenia not only delays functional recovery but also increases the risk of falls and reduces physical activity levels in stroke patients [9,10,11]. Sarcopenia, characterized by a loss of skeletal muscle mass and strength, exacerbates the functional impairments caused by stroke [12,13], and its effects are particularly detrimental when combined with stroke-induced muscle weakness and hemiplegia. In particular, sarcopenia, especially in patients with hemiplegia, can lead to further loss of strength on the non-paretic side, diminishing overall motor function [14]. This weakening of the musculoskeletal system can slow recovery by impairing balance, gait, and mobility, which are already compromised in stroke patients [11,15,16]. Moreover, reduced muscle mass can hinder patients’ ability to participate in and benefit from rehabilitation programs, which rely heavily on physical activity and strength training.

Exercise is a primary treatment for sarcopenia, with resistance and combined exercises being commonly suggested [17,18]. However, stroke patients require various forms of rehabilitation in addition to exercise. A previous study on stroke patients divided them into two groups based on whether their skeletal muscle mass index (SMI) and grip strength were reduced or not, specifically targeting stroke patients with a functional ambulation category (FAC) grade of 3 or higher, to evaluate the effects of rehabilitation [19]. Significant differences were observed between the two groups in the 6 min walk test and the timed up-and-go test after three weeks of rehabilitation. However, these studies had limitations, including a short intervention period and the inclusion of patients with relatively high functional status.

To address these limitations, this study focuses on acute stroke patients in the early recovery phase. Therefore, the aim of this study is to investigate the effect of sarcopenia on rehabilitation outcomes in acute stroke patients during the convalescent phase.

## 2. Materials and Methods

### 2.1. Study Procedure

**Figure 1:**
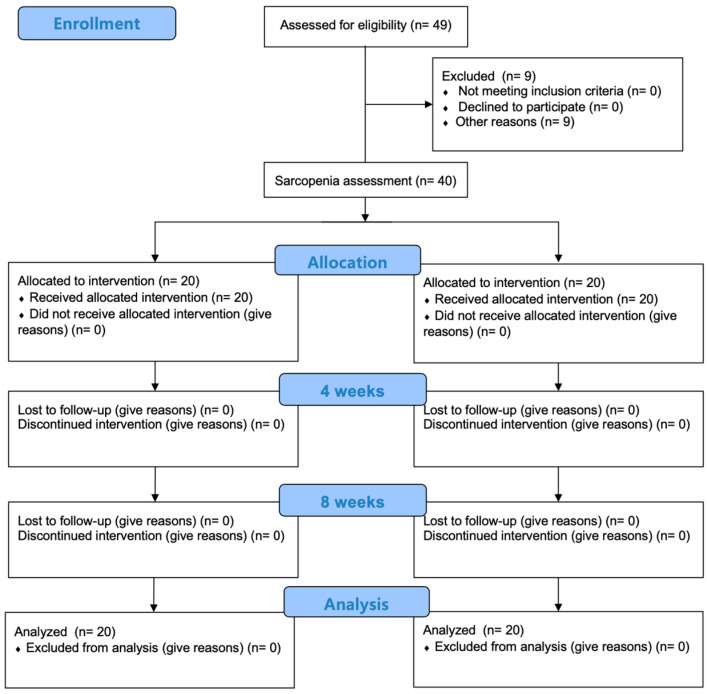
Study procedure.

This study was conducted with the approval of the institutional review board of Nambu University (IRB: 1041478-2023-HR-028), and it was registered in the Korea Registry of Clinical Trials (KCT0009795). It was carried out as a non-randomized controlled trial (Figure 1). Participants who agreed to participate, either by themselves or through their guardians, underwent an evaluation for sarcopenia. The participants were divided into a stroke with sarcopenia group and a stroke without sarcopenia group. Initially, 49 participants expressed interest in the study. However, as the study utilized quota sampling and the stroke with sarcopenia group had already met its quota of 20 participants, an additional 9 participants could not be enrolled and were therefore excluded from the study. All participants underwent evaluations before the intervention, after 4 weeks and after 8 weeks, and received the same convalescent rehabilitation. The assessors were blinded to the participants’ groupings, ensuring a single-blind study design.

### 2.2. Participants

This study was conducted on stroke patients admitted to the rehabilitation hospital in Gwangju. The inclusion criteria were patients admitted within 90 days after the onset of stroke or surgery. The exclusion criteria included patients with cardiovascular or orthopedic diseases that could pose a risk to or impact their participation in the study, patients with a level of consciousness below a drowsy state, and patients unable to undergo grip strength evaluation with at least one hand.

### 2.3. Diagnosis of Sarcopenia

Sarcopenia was assessed according to the 2019 criteria set by the Asian working group for sarcopenia [20]. A diagnosis of sarcopenia was made if both the SMI and grip strength were below the specified thresholds. The SMI thresholds are <7.0 kg/m^2^ for men and <5.7 kg/m^2^ for women. For grip strength, the cut-off values are <28 kg for men and <18 kg for women. The SMI was measured using bioelectrical impedance analysis with the BWA2.0 device (Inbody, Seoul, Republic of Korea) by a nurse. Evaluations were conducted either in a supine or standing position, depending on the participant’s condition, as recommended by the bioelectrical impedance analysis device. Grip strength was measured with a Jamar hydraulic hand dynamometer (B&L Engineering, Santa Ana, CA, USA) by an occupational therapist. If both hands could be tested, the higher value was recorded. If only one hand could be tested, the measurement from that hand was used. Sarcopenia was diagnosed by a rehabilitation physician.

### 2.4. Data Collection

#### 2.4.1. Muscle Strength

Manual muscle testing (MMT) was employed to evaluate the muscle strength of participants. MMT involves assessing twelve muscle groups in both the upper and lower extremities, including shoulder flexion, shoulder abduction, elbow flexion, elbow extension, wrist flexion, wrist extension, hip flexion, hip extension, knee flexion, knee extension, dorsiflexion, and plantar flexion. Each muscle group is rated on a scale where zero grade equals 1 point, trace grade equals 2 points, poor grade equals 3 points, fair grade equals 4 points, good grade equals 5 points, and normal grade equals 6 points. The individual scores for each muscle group are then added together to provide a total MMT score, with the highest possible score being 144 points [21]. The MMT assessments were conducted by a physical therapist. Although no separate reliability testing was conducted before data collection, MMT has been extensively validated and is commonly used to assess muscle strength in stroke patients [21].

#### 2.4.2. Balance

The Berg Balance Scale (BBS) was used to assess balance in stroke patients. The BBS consists of 14 items designed to evaluate balance through tasks related to sitting, standing, and postural changes. These tasks include sitting to standing, standing unsupported, sitting unsupported, transferring between chairs, standing with eyes closed, and standing on one leg. Each item is scored on a 5-point scale, ranging from 0 to 4. The total possible score is 56, with higher scores indicating better balance. The BBS assessments were conducted by a physical therapist. Although no separate reliability testing was conducted in this study, the BBS has been extensively validated and is known for its high reliability and validity in stroke patients [22,23].

#### 2.4.3. Gait

The functional ambulation category (FAC) was employed to assess gait in stroke patients. The FAC is a 6-level scale that categorizes a patient’s ability to walk based on the amount of assistance required. The levels range from 0 (the patient cannot walk or requires the help of two or more people) to 5 (the patient can walk independently on all surfaces without any supervision). It assesses functional mobility by focusing on how much assistance, if any, the patient needs to ambulate effectively. The FAC assessments were conducted by a physical therapist. No additional reliability testing was performed in this study; however, the FAC is widely recognized for its reliability and validity in assessing ambulation in stroke patients [24].

#### 2.4.4. Activities of Daily Living

The Modified Barthel Index (MBI) was used to assess the activities of daily living (ADL) in stroke patients. The MBI evaluates 10 ADL domains: feeding, bathing, grooming, dressing, bowel and bladder control, toilet use, chair transfer, ambulation, and stair climbing. Each domain is scored based on the level of independence, ranging from 0 to 5, with a total possible score of 100. Higher scores indicate greater independence in daily living activities. The MBI assessments were conducted by an occupational therapist. Although no separate reliability testing was performed in this study, the MBI has been validated in numerous studies and is recognized as a reliable tool for assessing ADL in stroke patients [25].

### 2.5. Convalescent Rehabilitation Program

In this study, both groups underwent a convalescent rehabilitation program. The program consisted of physical therapy and occupational therapy, conducted for 4 h a day, 7 days a week, for 8 weeks. Each group had two participants (a total of four) who required speech therapy; for these participants, the physical therapy time was reduced to accommodate speech therapy sessions.

### 2.6. Data Analysis

The sample size for this study was calculated using the G*Power (version 3.1) program, with an effect size of 0.40, a power of 0.80, and a significance level of 0.05, resulting in 36 participants. Considering a 10% dropout rate, the final sample size was determined to be 40 participants. Statistical analysis was performed using SPSS version 25 for Mac. Due to the data not meeting normality assumptions, non-parametric tests were adopted. The Mann–Whitney U test was used for between-group comparisons, and the Friedman test was used for within-group comparisons. Post hoc analysis within groups was conducted using Wilcoxon’s signed rank test. The general significance level was set at 0.05, and the post hoc analysis significance level was set at 0.0167.

## 3. Results

The general characteristics of the study participants are shown in Table 1. Significant differences between the two groups were observed only in the SMI and grip strength, which were used for the diagnosis of sarcopenia (*p* < 0.05).

The within-group comparison results are presented in Table 2. Significant differences in MMT were observed within both groups (*p* < 0.05). However, post hoc analysis within the stroke with sarcopenia group showed no significant differences. In the stroke without sarcopenia group, significant differences were found between weeks 1 and 4, 1 and 8, and 4 and 8 (*p* < 0.0167). For the BBS, both groups showed significant differences within the groups (*p* < 0.05). Post hoc analysis within the stroke with sarcopenia group revealed significant differences only between weeks 1 and 8 and 4 and 8 (*p* < 0.0167). In the stroke without sarcopenia group, significant differences were found between weeks 1 and 4, 1 and 8, and 4 and 8 (*p* < 0.0167). For the FAC, both groups had significant within-group differences (*p* < 0.05). Post hoc analysis within the stroke with sarcopenia group showed significant differences only between weeks 1 and 8 (*p* < 0.0167). In the stroke without sarcopenia group, significant differences were found between weeks 1 and 4, 1 and 8, and 4 and 8 (*p* < 0.0167). For the MBI, both groups demonstrated significant within-group differences (*p* < 0.05). Post hoc analysis within the stroke with sarcopenia group showed significant differences between weeks 1 and 4 and 1 and 8 (*p* < 0.0167). In the stroke without sarcopenia group, significant differences were found between weeks 1 and 4, 1 and 8, and 4 and 8 (*p* < 0.0167).

Between-group comparisons at 4 and 8 weeks revealed significant differences in multiple outcome measures (Table 3). After 4 weeks, the stroke with sarcopenia group showed smaller improvements in MMT, BBS, FAC, and MBI compared to the stroke without sarcopenia group, with statistically significant differences (*p* < 0.05). After 8 weeks, the differences in MMT, BBS, FAC, and MBI remained significantly smaller in the stroke with sarcopenia group compared to the stroke without sarcopenia group (*p* < 0.05).

## 4. Discussion

The purpose of this study was to investigate the effect of sarcopenia on the rehabilitation outcomes of acute stroke patients during the convalescent phase. Despite receiving intensive rehabilitation over the same period, the stroke with sarcopenia group showed less improvement in MMT, BBS, FAC, and MBI compared to the stroke without sarcopenia group. This finding provides novel insights into the detrimental effects of sarcopenia on functional recovery in stroke patients, even when intensive rehabilitation is provided. Our results emphasize the importance of identifying sarcopenia early in the rehabilitation process to implement tailored strategies aimed at improving recovery outcomes.

According to previous studies, reductions in SMI and grip strength negatively impact the functional recovery of subacute outpatient stroke patients. When comparing changes between the two groups, only patients without reductions in SMI or grip strength showed significant improvements in the 6 min walk test and the timed up-and-go test [19]. Our study produced similar results, indicating that providing more rehabilitation to stroke patients with lower functional levels than those in previous studies yielded comparable outcomes. Li et al. reported an association between sarcopenia and poor functional outcomes in acute stroke patients [7]. Sato et al. found that skeletal muscle mass in acute stroke patients affects swallowing function and independent eating activities [26]. Therefore, the presence of sarcopenia in acute stroke patients negatively impacts functional recovery, suggesting that stroke patients with sarcopenia may require additional or modified rehabilitation interventions to achieve comparable recovery outcomes to those without sarcopenia.

This study did not determine the extent to which the stroke with sarcopenia group overcame sarcopenia after 4 and 8 weeks. Nevertheless, since the stroke with sarcopenia group showed slower or less recovery compared to the stroke without sarcopenia group, additional or separate interventions are necessary to address sarcopenia. According to previous studies, rehabilitation aims to achieve functional improvements in stroke patients [27]. One study compared high-frequency and low-frequency groups performing sit-to-stand exercises to strengthen lower limb muscles within a rehabilitation program. It was reported that the frequency of sit-to-stand exercises was positively associated with improvements in sarcopenia and ADL in stroke patients undergoing rehabilitation [28]. Another study found that administering leucine-rich amino acid supplements for 8 weeks significantly improved ADL performance, muscle mass, and strength in elderly stroke patients with sarcopenia. Thus, additional nutritional supplementation during rehabilitation has been suggested [29]. Therefore, to overcome sarcopenia in stroke patients, new or additional rehabilitation programs may be necessary to replace existing ones.

This study has several limitations. First, it involved a small sample size from a single hospital, and the follow-up period of 8 weeks may have been insufficient to fully capture the long-term impact of sarcopenia on stroke recovery. Future research should include a larger sample and extend the follow-up period to confirm the long-term effects of sarcopenia, despite undergoing a standard rehabilitation program. Second, it was not possible to determine whether sarcopenia occurred before or after the stroke, nor could we identify the type of sarcopenia, as the participants were transferred to the rehabilitation hospital after receiving initial treatment for infarction or hemorrhage at other institutions. Third, the study did not include physical ability assessments since the participants were acute stroke patients. Additionally, differences between SMI measurements in supine and standing positions could exist, and participants’ measurement positions were not consistent throughout the study. Fourth, the rehabilitation programs of the participants were not uniformly supervised. Therefore, further research addressing these limitations is necessary.

## 5. Conclusions

Stroke with sarcopenia resulted in less improvement in functional abilities despite receiving the same period of intensive convalescent rehabilitation. Clinically, this study underscores the importance of early identification and tailored rehabilitation strategies for stroke patients with sarcopenia. These findings highlight the need for further research focused on stroke with sarcopenia.

## Figures and Tables

**Table 1:** General characteristics of the participants.

Variable	SWSG (*n* = 20)	SWOSG (*n* = 20)	*p*
Type of stroke (hemorrhage/infarct)	10/10	7/13	0.337
Vessel (large/small)	20/0	19/1	0.311
Territory (Carotid artery/ vertebro-basilar artery)	15/5	14/6	0.723
Hemiplegic side (Lt/Rt)	9/11	11/9	0.527
Sex (male/female)	8/12	9/11	0.749
Onset of stroke (day)	32.25 ± 21.48	32.20 ± 20.95	0.646
Age (years)	72.00 ± 10.48	66.45 ± 11.79	0.096
Height (cm)	160.15 ± 7.65	162.33 ± 6.40	0.272
Weight (kg)	57.64 ± 9.54	62.47 ± 10.19	0.175
MMSE (Score)	20.35 ± 5.88	24.00 ± 4.60	0.065
Skeletal muscle mass index (kg/m^2^)	5.42 ± 0.91	6.90 ± 0.88	<0.001 *
Grip strength (kg)	10.43 ± 8.50	17.50 ± 10.81	0.025 *
Pre-MMT (score)	106.35 ± 16.09	111.85 ± 12.80	0.357
Pre-BBS (score)	24.40 ± 17.53	26.80 ± 13.71	0.725
Pre-FAC (score)	1.55 ± 1.10	1.65 ± 0.99	0.692
Pre-MBI (score)	43.95 ± 22.41	48.75 ± 21.86	0.440

SWSG: stroke with sarcopenia group; SWOSG: stroke without sarcopenia group; MMSE: mini-mental state examination; MMT: manual muscle testing; BBS: Berg Balance Scale; FAC: functional ambulation category; MBI: Modified Barthel Index. * *p* < 0.05.

**Table 2:** Comparison of outcomes within groups.

Variable	Groups	4 Weeks	8 Weeks	χ2 (*p*)	1–4 Weeks Z (*p*)	1–8 Weeks Z (*p*)	4–8 Weeks Z (*p*)
MMT (score)	SWSG (*n* = 20)	107.95 ± 15.43	108.70 ± 15.42	7.538 (0.023 *)	−0.931 (0.352)	−1.980 (0.048)	−2.588 (0.100)
	SWOSG (*n* = 20)	116.20 ± 10.87	117.7 ± 11.73	24.000 (< 0.001 *)	−2.812 (0.005 ^†^)	−3.299 (0.001 ^†^)	−2.842 (0.004 ^†^)
BBS (score)	SWSG (*n* = 20)	25.85 ± 16.62	28.00 ± 15.66	12.035 (0.002 *)	−2.119 (0.034)	−2.617 (0.009 ^†^)	−2.613 (0.009 ^†^)
	SWOSG (*n* = 20)	37.10 ± 13.95	39.70 ± 13.30	29.681 (<0.001 *)	−3.434 (0.001 ^†^)	−3.622 (<0.001 ^†^)	−3.507 (<0.001 ^†^)
FAC (score)	SWSG (*n* = 20)	1.75 ± 1.16	2.00 ± 1.26	12.560 (0.002 *)	−2.000 (0.046)	−2.714 (0.007 ^†^)	−2.236 (0.025)
	SWOSG (*n* = 20)	2.5 ± 1.19	3.10 ± 1.33	25.107 (<0.001 *)	−2.942 (0.003 ^†^)	−3.482 (<0.001 ^†^)	−3.464 (0.001 ^†^)
MBI (score)	SWSG (*n* = 20)	47.10 ± 21.15	49.10 ± 20.96	15.500 (<0.001 *)	−2.439 (0.015 ^†^)	−2.731 (0.006 ^†^)	−2.099 (0.036)
	SWOSG (*n* = 20)	59.25 ± 16.77	64.10 ± 17.69	34.060 (<0.001 *)	−3.728 (<0.001 ^†^)	−3.725 (<0.001 ^†^)	−3.186 (0.001 ^†^)

SWSG: stroke with sarcopenia group; SWOSG: Stroke without sarcopenia group; MMT: manual muscle testing; BBS: Berg Balance Scale; FAC: functional ambulation category; MBI: Modified Barthel Index. * *p* < 0.05 and ^†^ *p* < 0.0167.

**Table 3:** Comparison of outcomes between groups.

Difference Value	SWSG (*n* = 20)	SWOSG (*n* = 20)	*p*
MMT difference after 4 weeks	1.60 ± 5.17	4.35 ± 6.63	0.027 *
MMT difference after 8 weeks	2.35 ± 5.26	5.85 ± 7.13	0.038 *
BBS difference after 4 weeks	1.45 ± 3.63	10.30 ± 12.22	0.004 *
BBS difference after 8 weeks	3.60 ± 5.83	12.90 ± 12.86	0.006 *
FAC difference after 4 weeks	0.20 ± 0.41	0.85 ± 1.04	0.014 *
FAC difference after 8 weeks	0.45 ± 0.60	1.45 ± 1.19	0.003 *
MBI difference after 4 weeks	3.15 ± 5.12	10.50 ± 11.69	0.009 *
MBI difference after 8 weeks	5.15 ± 7.49	15.35 ± 14.49	0.007 *

SWSG: stroke with sarcopenia group; SWOSG: stroke without sarcopenia group; MMT: manual muscle testing; BBS: Berg Balance Scale; FAC: functional ambulation category; MBI: Modified Barthel Index. * *p* < 0.05.

## Data Availability

The data can be requested from the corresponding author and will be released on reasonable request.
